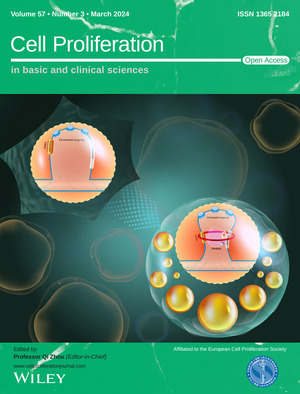# Featured Cover

**DOI:** 10.1111/cpr.13628

**Published:** 2024-03-01

**Authors:** Xiaokai Li, Sha Zeng, Li Chen, Yu Zhang, Xuemin Li, Biwei Zhang, Duo Su, Qinjiao Du, Jiaman Zhang, Haoming Wang, Zhining Zhong, Jinwei Zhang, Penghao Li, Anan Jiang, Keren Long, Mingzhou Li, Liangpeng Ge

## Abstract

The cover image is based on the Original Article *An intronic enhancer of Cebpa regulates adipocyte differentiation and adipose tissue development via long‐range loop formation* by Xiaokai Li et al., https://doi.org/10.1111/cpr.13552.